# On Feeling Torn About One’s Sexuality: The Effects of Explicit–Implicit Sexual Orientation Ambivalence

**DOI:** 10.1177/0146167214539018

**Published:** 2014-09

**Authors:** Ben Windsor-Shellard, Geoffrey Haddock

**Affiliations:** 1 Cardiff University, UK

**Keywords:** explicit–implicit ambivalence, information processing, sexual orientation

## Abstract

Three studies offer novel evidence addressing the consequences of explicit–implicit sexual orientation (SO) ambivalence. In Study 1, self-identified straight females completed explicit and implicit measures of SO. The results revealed that participants with greater SO ambivalence took longer responding to explicit questions about their sexual preferences, an effect moderated by the direction of ambivalence. Study 2 replicated this effect using a different paradigm. Study 3 included self-identified straight and gay female and male participants; participants completed explicit and implicit measures of SO, plus measures of self-esteem and affect regarding their SO. Among straight participants, the response time results replicated the findings of Studies 1 and 2. Among gay participants, trends suggested that SO ambivalence influenced time spent deliberating on explicit questions relevant to sexuality, but in a different way. Furthermore, the amount and direction of SO ambivalence was related to self-esteem.

The simultaneous experience of positive and negative feelings toward an attitude object characterizes *attitudinal ambivalence* (DeMarree, Wheeler, Briñol, & Petty, 2014; Maio & Haddock, 2010). Ambivalence is common, whether it refers to racial attitudes, interpersonal attitudes, or even people’s self-evaluations (e.g., Haddock & Gebauer, 2011; Jordan, Logel, Spencer, Zanna, & Whitfield, 2009; van Harreveld, van der Pligt, & de Liver, 2009). More recently, research has become concerned with the effects of *explicit–implicit ambivalence* (e.g., Briñol, Petty, & Wheeler, 2006; Petty & Briñol, 2009; Rydell, McConnell, & Mackie, 2008). This reflects a discrepancy that occurs between explicit (i.e., direct) and implicit (i.e., indirect) evaluations of an attitude object. The present research is interested in understanding the impact of explicit–implicit ambivalence in people’s thoughts and feelings about their sexual orientation (SO).

Generally, research has shown there to be negative psychological consequences associated with explicit–implicit ambivalence. For example, a larger explicit–implicit discrepancy in self-esteem is associated with greater self-doubt, depression, and loneliness (Briñol et al., 2006; Creemers, Scholte, Engels, Prinstein, & Wiers, 2012). Such negative consequences have been suggested to result from explicit–implicit ambivalence producing an internal state of discomfort, which is then used by individuals to interpret their well-being (Rydell & Durso, 2012; Rydell et al., 2008). Furthermore, ambivalence is described as a state of aversion and interference between newly acquired attitudes and old attitudes retained in memory, implying that ambivalence is a state individuals are motivated to reduce (Petty, Tormala, Briñol, & Jarvis, 2006; van Harreveld et al., 2009).

## The Impact of Ambivalence on Information Processing

Considerable research has addressed the consequences of attitudinal ambivalence on information processing. The majority of this work has addressed the implications of *explicit* attitudinal ambivalence, with research showing that explicit ambivalence results in more systematic processing of ambivalence-related information. For example, Maio, Bell, and Esses (1996) found that individuals who were ambivalent toward Asians allocated greater attention to a strong versus weak persuasive message about immigration compared with non-ambivalent individuals. More recently, Clark, Wegener, and Fabrigar (2008) found that ambivalent individuals processed more deeply pro-attitudinal messages, perceiving that such messages would decrease ambivalence. These studies imply that ambivalent individuals engage in more processing of relevant information to reduce the experienced conflict.

Research has also shown *explicit–implicit ambivalence* to result in systematic processing of ambivalence-relevant information. Briñol et al. (2006) had participants complete explicit (E) and implicit (I) measures of shyness. Participants’ scores on each measure were standardized to calculate the *amount* of ambivalence (the magnitude of the E–I difference) and the *direction* of ambivalence (whether the explicit score or the implicit score is higher). These researchers found a main effect of the *amount* of ambivalence on processing, with larger discrepancies between explicit and implicit shyness measures resulting in a more careful consideration of shyness-related information. Similarly, Rydell et al. (2008) found that, after creating explicit and implicit attitudes, ambivalent participants were persuaded after the elaborate processing of relevant strong arguments (but not relevant weak arguments). Taken together, these studies suggest that explicit–implicit ambivalence results in greater attention to information that is relevant to the ambivalence. One aim of the present research is to investigate whether explicit–implicit ambivalence in the context of an individual’s thoughts toward their SO has similar consequences.

## Explicit–Implicit Sexual Orientation Ambivalence

SO concerns an individual’s sexual attraction, behavior, and identity (e.g., as a straight/gay individual; Savin-Williams, 2006). Given that some individuals conceal their SO (Legate, Ryan, & Weinstein, 2011; Meyer, 2003), explicit measures may not always be informative. Consequently, there has been interest in developing implicit measures of SO that seek to avoid presentational biases. Building upon techniques such as the Implicit Association Test (IAT; Greenwald, McGhee, & Schwartz, 1998), indirect measures of SO typically involve a reaction time task that considers the strength of an individual’s association regarding their sexuality.

The importance of using *both* explicit and implicit measures of SO has become evident in light of research assessing the consequences of divergence between responses obtained from such measures. In one study, Weinstein et al. (2012) considered whether the relationship between explicitly and implicitly measured SO was moderated by the amount of experienced autonomy (in the context of autonomy controlling versus non-controlling parents). In this study, which used a single-item explicit measure of SO and an adapted evaluative priming task (Fazio, Jackson, Dunton, & Williams, 1995), it was found that when straight individuals perceived high parental autonomy control, there was no relation between scores on the explicit and implicit measures of SO. However, when individuals perceived low parental autonomy control, scores on the explicit and implicit measures of SO were convergent. In addition, participants who self-reported as more straight on the explicit measure relative to the implicit measure (i.e., showed a relative gay orientation on the implicit measure) expressed more homophobic attitudes than other respondents. These findings imply that the relationship between explicit and implicit measures of SO is associated with important consequences.

## Aims of the Current Research

In this research, three studies investigated the implications of explicit–implicit SO ambivalence by assessing individual differences in the *amount* and *direction* of ambivalence. On the basis of evidence demonstrating that explicit–implicit attitudinal ambivalence results in more systematic processing of ambivalence-relevant information (Briñol et al., 2006; Rydell et al., 2008), Study 1 investigated whether greater explicit–implicit SO ambivalence among a sample of straight-identified females is associated with greater deliberation when responding to direct questions about one’s SO. Study 2 investigated the degree to which the effects in Study 1 are robust by considering how individuals respond to information relevant to SO. Study 3 extended the work by using a more diverse sample of straight and gay male and female participants. As a secondary aim, in light of findings regarding the association between SO and mental health (e.g., Haas et al., 2011; Meyer, 2003), Study 3 investigated whether explicit–implicit SO ambivalence is associated with well-being.

## Study 1: Sexual Orientation Ambivalence and the Deliberation of Direct Questions About Sexual Orientation

Unlike previous research on the effects of explicit–implicit ambivalence, this study is not interested in the *persuasion* of ambivalent individuals. Instead, we investigated whether explicit–implicit SO ambivalence was related to the amount of time spent thinking about one’s sexuality. Research has found that ambivalence is associated with longer response times to attitude-relevant questions, as a consequence of requiring time to consider conflicting attitudinal attributes (e.g., Bassili, 1996; van Harreveld, van der Pligt, de Vries, Wenneker, & Verhue, 2004). As such, our outcome variable was the amount of time participants spent deliberating direct questions about their SO. Using this conceptualization, individuals who took longer to respond to direct questions about their SO were considered to be those who experienced more conflict. On the basis of previous research, we expected individuals with larger explicit–implicit discrepancies to spend longer responding to direct questions about their SO.

### Method

#### Participants

Fifty-eight straight-identified female Cardiff University students participated for course credit (*M*_age_= 20.23 years, *SD* = 2.31 years).

#### Materials

##### Explicit measure of SO

Five items assessed opposite-sex attraction and behavior (e.g., I find men attractive; I have sex with men; α = .66), and five items assessed same-sex attraction and behavior (e.g., I find women attractive; I have sex with women; α = .64). Participants rated their agreement with each item on a nine-point scale (1 = *definitely not reflective of me*; 9 = *definitely reflective of me*). Response time for each item was assessed to give an index of time spent thinking about aspects of SO.

##### Implicit measure of sexual orientation

The implicit measure of SO was a personalized IAT (see Han, Olson, & Fazio, 2006). In five stages, this task assessed the strength of the association between an individual, their SO, and comparison categories (another person, not the participant’s SO). Reliability was computed using split-half reliability analysis between odd and even trials (Karpinski & Steinman, 2006) and was acceptable (adjusted *r* = .68).

In the first stage (10 trials), using two response keys (*Me* on the left side of the keyboard [key E], and *Not me* on the right [key I]), participants categorized words that were representative of themselves or a fictitious character. Representative words corresponded to personal information (e.g., first name, surname, place of birth) specified by the participant at the beginning of the study.

In Stage Two (10 trials), using two response keys (Gay [E] and Straight [I]) participants classified pictures of either gay female couples or straight couples. In all, there were five pictures of gay female couples and five pictures of straight couples (taken from publicly available sources).

Stage Three (20 trials) contained the first set of critical trials where the category labels from Stages One and Two were combined. One response key (Gay *or* Me; [E]) was used to categorize words that were representative of the participant *or* pictures of gay couples. The other response key (Straight *or* Not me; [I]) was used to categorize words that were not representative of the participant *or* pictures of straight couples.

In Stage Four (10 trials), participants repeated Stage One. However, the response keys of the category labels changed positions.

The final stage (Stage Five) contained the second set of (20) critical trials, this time used to assess the automatic association between a participant and *their* self-identified SO. One response key (Gay *or* Not me; [E]) was used to categorize words that were *not* representative of the participant *or* pictures of gay couples. The other response key (Straight *or* Me; [I]) was used to categorize words that were representative of the participant *or* pictures of straight couples.

##### Computation of IAT effect

IAT effects were computed on the basis of a D’ score (Greenwald, Nosek, & Banaji, 2003). Prior to computing this index, any response time greater than 10,000 ms is deleted, in addition to discarding cases where more than 10% of scores are less than 300 ms (no violations occurred in the study).

##### Explicit–implicit discrepancy

To investigate the impact of explicit–implicit SO ambivalence on the amount of time taken to think about sexuality, we calculated parameters of the amount and the direction of ambivalence, following the procedure outlined by Briñol et al. (2006). These values were derived by calculating the difference between standardized scores on the explicit and implicit measures of SO. The *amount* of SO ambivalence concerns the absolute value of this difference, such that the greater the value from zero, the greater the discrepancy between scores on the explicit and implicit measures. The *direction* of SO ambivalence concerns the relative positivity or negativity of the standardized explicit–implicit difference. When a negative value was calculated (indicating that an individual had a *lower* score on the explicit measure of SO relative to the implicit measure [E < I]), a dummy code of −1 was used. When a positive value was calculated (indicating that an individual had a *higher* score on the explicit measure of SO relative to the implicit measure [E > I]), a dummy code of +1 was used. Thus, for this self-reported straight sample, there were two directions of SO ambivalence: (a) those who reported being *less straight* on the explicit measure of SO relative to the implicit measure (E < I), and (b) those who reported being *more straight* on the explicit measure of SO relative to the implicit measure (E > I).

#### Procedure

The study was conducted using DirectRT (Jarvis, 2008). Participants completed the explicit measures of SO prior to completing the implicit measure of SO.^1^ Participants then completed other measures not relevant to the current discussion.

### Results

#### Descriptive statistics

##### Sexual orientation measures

As would be expected in our self-reported straight female sample, the explicit measure of SO showed a significantly stronger preference for men (*M* = 8.60, *SD* = .57) over women (*M* = 1.60, *SD* = .69), *t*(57) = 52.87, *p* < .0001. The implicit measure of SO showed an IAT effect indicative of a straight SO (*M*D’ = .66, *SD* = .41). This value was significantly different from zero, *t*(57) = 12.06, *p* < .0001, indicating that the test measured a difference in valence between the critical blocks.

The correlation between the explicit and implicit measures of SO was not significant, *r* = .06, *ns*.^2^

#### The impact of SO ambivalence on the time spent on explicit questions relating to sexuality

In a regression model, we used the amount and the direction of ambivalence and their interaction as predictor variables.^3^ The outcome variable was the mean reaction time of all items on the explicit measure of SO (as the same-sex and opposite-sex item reaction times were highly correlated, *r* = .63). The analysis revealed a significant main effect of the amount of SO ambivalence, β = .31, *t*(54) = 2.73, *p* = .01. This effect indicates that greater ambivalence was associated with spending more time thinking about sexuality and is consistent with other work on the effects of explicit–implicit ambivalence.

**Figure 1.**
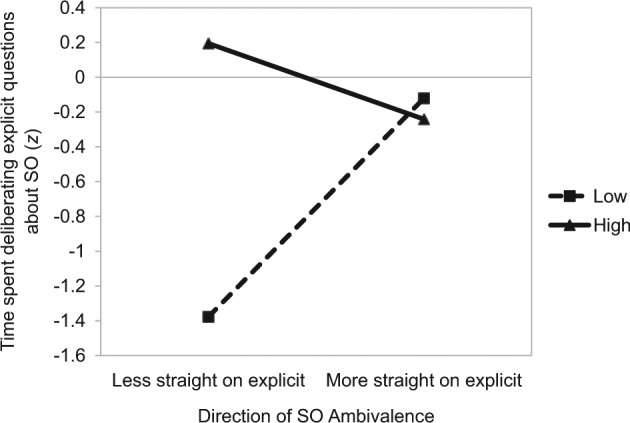
Study 1: The impact of the amount (separate lines) and direction (*x* axis) of SO ambivalence on time spent deliberating explicit questions on one’s sexuality. *Note.* SO = sexual orientation.

Interestingly, the main effect was qualified by a significant amount by direction interaction, β = −.58, *t*(54) = −3.18, *p* = .002 (see Figure 1). This interaction shows that the distinction between individuals with high and low amounts of ambivalence was present when individuals reported being l*ess straight* on the explicit measure of SO relative to the implicit measure.^4^ Specifically, those with high amounts of SO ambivalence spent significantly more time deliberating their SO relative to those with low amounts of SO ambivalence, β = .79, *t*(54) = 5.07, *p* < .0001. However, when considering those individuals who reported being *more straight* on the explicit measure of SO relative to the implicit measure, there was no difference in deliberation as a function of the amount of SO ambivalence, β = −.06, *t* < 1.

### Discussion

The primary aim of this study was to ascertain whether greater SO ambivalence results in more thinking about one’s SO. Consistent with extant research that has investigated the effects of explicit–implicit ambivalence (Briñol et al., 2006; Rydell et al., 2008), we found a significant main effect of the *amount* of explicit–implicit SO ambivalence on deliberation. As a consequence of needing to consider a range of conflicting attributes (van Harreveld et al., 2004), individuals with larger discrepancies between the explicit and implicit measures of SO generally spent more time responding to direct questions about their sexuality.

Interestingly, this main effect was qualified by an unexpected amount by direction interaction. There was a clear distinction between those with low and high amounts of SO ambivalence *only* when individuals reported being less straight on the explicit measure of SO relative to the implicit measure. In this directional context, those with high SO ambivalence took significantly more time to respond to the explicit questions of SO than those with low SO ambivalence. However, among individuals who reported being *more straight* on the explicit measure of SO relative to the implicit measure, the amount of ambivalence did not affect the time spent deliberating SO.

## Study 2: Sexual Orientation Ambivalence and Its Impact on Information Processing

The results of Study 1 provide important insights regarding explicit–implicit SO ambivalence. Consistent with past research, greater explicit–implicit ambivalence was associated with longer deliberation of relevant information. However, Study 1 also revealed an unexpected interaction between the amount and direction of SO ambivalence. As such, one aim of Study 2 was to assess the replicability of the findings.

In addition, Study 2 used another type of outcome measure to test the robustness of the pattern of findings. Despite arguments advocating the utility of a response time measure of deliberation in the context of ambivalence (e.g., van Harreveld et al., 2004), we believed it important and necessary to use a strategy that directly separates ambivalence from response times. To do this, Study 2 incorporated a paradigm where participants read information supporting gay marriage that contained information that was either high in relevancy to SO (e.g., links to equality) or low in relevancy to SO (e.g., benefits on waiting times of civil marriage ceremonies). We expected that an individual’s SO ambivalence would influence their subsequent deliberation after reading ambivalence-relevant information. To the extent that Study 1’s findings reflect differences in deliberation, we expected the amount of elaboration articulated by participants in response to highly relevant information to follow the pattern we observed on the response latency measure. However, in response to less relevant information, we expected an individual’s SO ambivalence not to impact elaboration.

Elaboration was assessed by having participants indicate the thoughts that came to mind after reading the information about gay marriage. This technique is a well-established measure of the extent of information processing (Cacioppo, von Hippel, & Ernst, 1997; Greenwald, 1968). Moreover, ambivalent individuals generate more thoughts in reaction to ambivalence-relevant information (Jonas, Diehl, & Brömer, 1997), which has been found to reduce ambivalence (Nordgren, van Harreveld, & van der Pligt, 2006). As such, the amount of elaboration is a good indicator of attempts to resolve ambivalence.

As a secondary measure, we also considered participants’ post-message attitude favorability toward the introduction of gay marriage. However, because of the strength of positive feeling on this issue, we were not certain that this measure would elicit different effects as a function of ambivalence and the relevance of the presented information.

### Method

#### Participants

One hundred fifteen self-identified straight females (*M*_age_ = 19.12 years, *SD* = 1.57) participated for course credit.

#### Materials

##### Sexual orientation and SO ambivalence

The explicit and implicit measures of SO were those used in Study 1. The explicit measure of SO was coded according to opposite-sex (α = .67) and same-sex attraction (α = .55). The implicit measure of SO orientation was reliable (adjusted *r* = .68). As described in Study 1, SO ambivalence was conceptualized in terms of individual differences in the amount of explicit–implicit discrepancy (the absolute difference between the standardized scores on the explicit and implicit measures of SO) and the direction of the discrepancy (dummy code of +1 or −1 according to the valence of the non-absolute difference between the standardized scores on the explicit and implicit measures of SO).

##### Manipulation of topic relevance

Participants read one of two editorials on the introduction of gay marriage that varied in terms of relevance to SO. The *high relevance* editorial contained information that clearly was related to SO. Specifically, this editorial referred to the views of a gay rights charity, research that supported the robustness of same-sex families, reduction of sexual stigma, and evidence that stipulated the detrimental psychological effects of denying equality to same-sex couples. The *low relevance* editorial contained information that clearly was not related to SO. Specifically, this editorial referred to the views of a registry office spokesperson, anticipated monetary gains for the government, and improved inheritance tax rights.^5^ The effects of the manipulation were distinguished by assigning a dummy code of +1 to the high relevance editorial and a dummy code of −1 to the low relevance editorial.

##### Post-message attitude toward the introduction of gay marriage

Participants were asked “On the basis of the article, how favorable is your attitude towards the introduction of gay marriage?” Participants responded using a nine-point scale (1 = very unfavorable; 9 = very favorable).

##### Measure of elaboration

To measure elaboration, participants reported their thoughts in response to the information they read. After each listed thought, individuals reported its valence. Our outcome variable was the level of elaboration (in words) reported in their responses.^6^

#### Procedure

The study was conducted using DirectRT (Jarvis, 2008). Participants completed the explicit measure of SO prior to completing the implicit measure of SO. Subsequently, participants read information on the introduction of gay marriage prior to reporting their thoughts and attitudes in response to the information read.

### Results

#### Descriptive statistics

##### Sexual orientation measures

As would be expected in our self-reported straight female sample, the explicit measure of SO showed a significantly stronger preference for men (*M* = 8.66, *SD* = .67) over women (*M* = 1.70, *SD* = .82), *t*(114) = 67.67, *p* < .0001. The implicit measure of SO showed an IAT effect indicative of a straight SO (*M*D’ = .58, *SD* = .38). This value was statistically different from zero, *t*(114) = 16.44, *p* < .0001, indicating that the measure was assessing a difference in valence between the critical blocks.

Within this study, responses on the explicit and implicit measures of SO showed a small but significant correlation, *r* = .18, *p* = .05.

#### The impact of SO ambivalence on the time spent on explicit questions relating to sexuality

**Figure 2.**
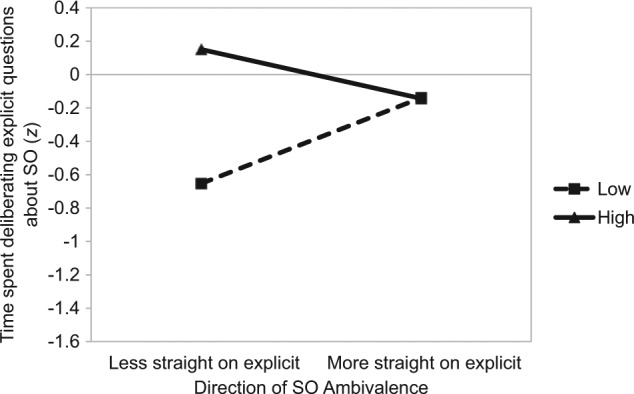
Study 2: The impact of the amount (separate lines) and direction (*x* axis) of SO ambivalence on time spent deliberating explicit questions on one’s sexuality. *Note.* SO = sexual orientation.

Consistent with Study 1, a regression analysis revealed that greater SO ambivalence was (marginally) associated with longer deliberation in response to explicit questions about sexuality, β = .17, *t*(111) = 1.80, *p* = .08. This effect was once again qualified by the amount by direction interaction, β = −.26, *t*(111) = −1.81, *p* = .07 (see Figure 2). Like Study 1, when self-identified straight females reported an SO on the explicit measure that was less straight than that stipulated by the implicit measure, individuals with high SO ambivalence took longer to respond to direct sexuality questions compared with those with low SO ambivalence, β = .40, *t*(111) = 3.00, *p* = .003. However, for individuals who reported an SO on the explicit measure that was more straight than that stipulated by the implicit measure, the amount of ambivalence had no effect, β = .00, *t* < 1. Together, these findings replicate those obtained in Study 1.

#### The impact of SO ambivalence on elaboration

In a regression model, we included the amount and the direction of SO ambivalence, information relevance (high/low), and the respective interactions as the independent variables. The outcome variable was the elaboration of respondents’ thoughts. The analysis revealed a significant amount by direction interaction, β = −.39, *t*(107) = −2.68, *p* = .008. Overall, individual differences in SO ambivalence related to post-message thought elaboration in a pattern that is identical to that observed with the response latency outcome.

**Figure 3.**
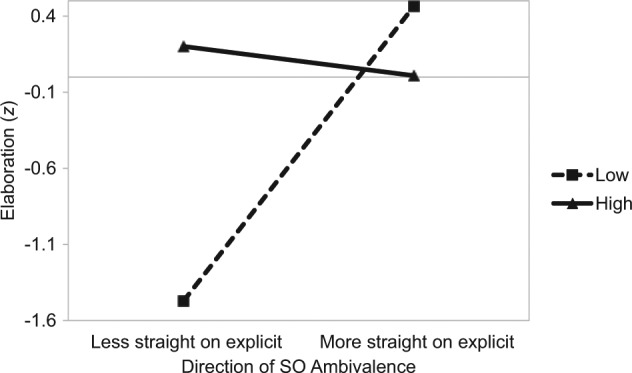
Study 2: The impact of the amount (separate lines) and direction (*x* axis) of SO ambivalence on elaboration after reading highly relevant information. *Note.* SO = sexual orientation.

However, this effect was qualified by a significant three-way interaction, β = −.29, *t*(107) = −1.98, *p* = .05, such that the interaction between the amount and the direction of SO ambivalence was moderated by information relevancy. Among participants who read highly relevant information, there was a significant difference in elaboration as a function of the amount and direction of SO ambivalence, β = −.60, *t*(53) = −3.25, *p* = .002 (see Figure 3). As with the response latency outcome, this interaction revealed two key findings. First, among individuals who reported being less straight than in the explicit measure of SO than that stipulated by the implicit measure, those with high SO ambivalence elaborated more compared with those with low SO ambivalence β = .84, *t*(53) = 3.67, *p* = .001. Second, for those who reported being more straight on the explicit measure of SO than that stipulated by the implicit measure, there was no difference in elaboration as a function of the amount of SO ambivalence, β = −.23, *t* < 1. This pattern parallels the response time findings and offers convergent evidence that deliberation about sexuality differs as a function of the amount and direction of SO ambivalence.

When individuals were presented with less relevant information regarding gay marriage, the main effect of the amount of ambivalence on elaboration was non-significant (*p >* .90), in addition to the interaction between the amount and direction of SO ambivalence (*p* > .60).^7^

#### The impact of SO ambivalence on post-message attitude toward the introduction of gay marriage

In a regression model, we included the amount and the direction of SO ambivalence, information relevance, and the respective interactions as the independent variables, with attitude toward gay marriage as the outcome variable. As expected, attitudes toward gay marriage were very positive (*M* = 7.89; *SD* = 1.36), suggesting a ceiling effect. That said, the analysis revealed a significant main effect of the amount of SO ambivalence, β = .23, *t*(107) = 2.40, *p* = .02. Overall, greater SO ambivalence was associated with more favorable attitudes toward gay marriage. This main effect was qualified by a significant interaction between the amount of SO ambivalence and information relevance, β = .29, *t*(107) = 2.00, *p* = .05. The amount of ambivalence only affected attitudes among participants who were presented with the highly relevant information supporting gay marriage, β = .36, *t*(53) = 2.77, *p* = .008. Ambivalence did not impact attitudes among individuals presented with low relevance information supporting gay marriage, β = .05, *t*(54) < 1.

### Discussion

Study 2 investigated the replicability and robustness of the findings observed in Study 1. When considering response latency to direct questions about sexuality, the results of this study replicated those found in Study 1. Specifically, there was a main effect of the amount of SO ambivalence that was qualified by an interaction between the amount and direction of SO ambivalence. The interaction pattern once again revealed that among individuals who reported being *less straight* on the explicit measure of SO relative to the implicit measure, those with high SO ambivalence took significantly longer to respond to the direct questions when compared with those with low SO ambivalence. However, when considering those who reported being *more straight* on the explicit measure of SO relative to the implicit measure, there was no observable difference in response time as a function of the amount of ambivalence.

The robustness of the effects on the response time measure was assessed by adopting a paradigm that provided a different means to assess the deliberation of ambivalence-relevant information. In the study, participants read high or low relevance information for the introduction of gay marriage. Subsequently, we measured thought elaboration in response to the information. Overall, the amount of elaboration followed a pattern consistent with the response time measure (an effect moderated by information relevance). Among individuals who read highly relevant information, the findings on the elaboration measure yielded effects that converged with the response time measure, and provide more direct evidence that the response time findings reflect deliberation. When considering individuals who reported being *less straight* on the explicit measure of SO than the implicit measure, there was a significant difference in elaboration as a function of the amount of ambivalence. Consistent with research that has stipulated thought listing to attenuate the negative effects of ambivalence (Nordgren et al., 2006), this finding implies that the amount of ambivalence in this directional context influences deliberation in an attempt to reduce the associated conflict. In other words, we suggest that individuals with high amounts of SO ambivalence in this directional context experienced more conflict and sought to resolve it by elaborating upon the information in more detail.

Conversely, when considering individuals who reported being *more straight* on the explicit measure of SO relative to the implicit measure, there was no difference in the elaboration of thoughts as a function of the amount of ambivalence. We believe that this finding can be explained by the implications associated with this direction of SO ambivalence. Specifically, this group of self-identified straight individuals may be concealing some elements of same-sex attraction that are identified in the implicit measure of SO. These associations are in conflict with self-identified SO; the mere fact that this conflict exists, as opposed to its magnitude, may be sufficient to elicit the effects associated with ambivalence and hence, motivation to reduce these effects.

Another interesting aspect of the results is that post-message attitudes did not fully converge with the pattern of findings on the other measures. Here, after reading highly relevant information, individuals with high amounts of SO ambivalence had more positive attitudes toward the introduction of gay marriage than those with low amounts of ambivalence (an effect that was moderated by information relevance).

## Study 3: Sexual Orientation Ambivalence and Its Impact on Deliberation and Well-Being in Straight and Gay Individuals

The results of Studies 1 and 2 demonstrate a convergent and novel pattern of findings. In both studies, we found an interaction between the amount and the direction of SO ambivalence on the time spent deliberating explicit questions on SO. In addition, Study 2 showed the robustness of this effect by demonstrating its generalizability to a different paradigm that disentangles response time from ambivalence.

Study 3 sought to extend these findings in a number of ways. First, this study used a more diverse sample of straight *and* gay men *and* women. To our knowledge, this is the first study of its kind on the nature of SO ambivalence on information processing as a function of participants’ SO, and as such, we do not make predictions about possible deliberation differences for gay participants.

Second, we also investigated the impact of explicit–implicit SO ambivalence on individuals’ well-being. There is evidence that concealment of SO has negative psychological consequences (Hatzenbuehler, 2009; Meyer, 2003). In addition, individuals who conceal their SO have difficulty in forming a positive identity of their SO (Frable, Wortman, & Joseph, 1997). As such, it is plausible that self-identified straight individuals who experience implicit evaluations of their SO that are in conflict with self-reported identification may experience negative outcomes.

Third, research has generally found gay individuals to be at greater risk for mental health problems relative to straight individuals (e.g., Haas et al., 2011; King et al., 2003, 2008; Meyer, 2003). Furthermore, explicit and implicit anti-gay attitudes among gay individuals and hence, *self-stigmatization*, are important predictors of psychological distress (Hatzenbuehler, Dovidio, Nolen-Hoeksema, & Phills, 2009). Therefore, Study 3 also investigated the impact of holding different explicit and implicit evaluations of SO on well-being. Accordingly, we included explicit and implicit measures of individuals’ *affect* about their SO, and considered whether such affective responses are associated with well-being.

### Method

#### Participants

Seventy self-identified straight participants (49 females; *M*_age_ = 20.04 years, *SD* = 2.15 years) and 48 self-identified gay participants (12 females; *M*_age_ = 31.65 years, *SD* = 12.38 years) participated for course credit or £5.

#### Materials

##### Sexual orientation and SO ambivalence

We used the same explicit and implicit measures of SO as outlined earlier. The explicit measure of SO was coded according to opposite-sex attraction (α = .97) and same-sex attraction (α = .95). Given the more diverse nature of the sample, the implicit measure was altered so that male participants saw gay male couples and female participants saw gay female couples. The reliability of the implicit measure was high (adjusted *r* = .92).

As in Studies 1 and 2, SO ambivalence was conceptualized in terms of individual differences in the amount of explicit–implicit discrepancy (the absolute difference between the standardized scores on the explicit and implicit measures of SO) and the direction of the discrepancy (dummy code of +1 or −1 according to the valence of the non-absolute difference between the standardized scores on the explicit and implicit measures of SO). For gay participants, the direction of SO ambivalence reflected: (a) those who reported being *less gay* on the explicit measure of SO relative to the implicit measure (E < I), and (b) those who reported being *more gay* on the explicit measure of SO relative to the implicit measure (E > I).

##### Measures of self-esteem

The study used explicit (ESE) and implicit (ISE) measures of self-esteem. The explicit measure was the Single Item Self-Esteem measure (Robins, Hendin, & Trzesniewski, 2001). Participants indicated their agreement to the statement “I have high self-esteem” (1 = *does not apply at all*; 9 = *applies completely*). This measure is highly correlated with multi-item measures and has temporal stability (Robins et al., 2001).

The implicit measure was the Single Item Name-Liking measure (Gebauer, Riketta, Broemer, & Maio, 2008). Participants were asked “How much do you like your name, in total?” (1 = *not at all*; 9 = *very much*). This measure has high test–retest reliability and is correlated with other indirect measures of self-esteem (Gebauer et al., 2008).

##### The importance of sexual orientation as a component of the self

This measure was based on the *centrality* facet of Cameron’s (2004) social identity measure. Seven items were adapted to SO (α = .84). Sample items include “I often think about the fact that I am gay/straight” and “Overall, being gay/straight has very little to do with how I feel about myself” (reverse scored; orientation frames derived from self-reported responses). The items were rated on a nine-point scale (1 = *strongly disagree*; 9 = *strongly agree*).

##### Explicit measure of affect toward one’s sexual orientation

This measure was based on the *affect* facet of Cameron’s (2004) social identity measure. Five items were adapted to SO (α = .75). Sample items include “In general, I am glad to be gay/straight” and “I often regret that I am gay/straight” (reverse scored). The items were rated on a nine-point scale (1 = *strongly disagree*; 9 = *strongly agree*).

##### Implicit measure of affect toward one’s sexual orientation

This measure was an IAT that assessed the strength of the association between an individual’s SO and positive/negative affect words. Like the SO IAT, Stages One (10 trials) and Two (10 trials) were simple categorization tasks. In Stage One, participants classified pictures that were representative of their sexual orientation or not representative of their sexual orientation. The pictures, taken from publically available sources, were pictures of straight or gay couples, and were different from those used in the SO IAT. In Stage Two, participants classified words as either “positive” (e.g., happiness, warmth) or “negative” (e.g., corpse, vomit). In Stage Three (20 trials), both pictures and words were presented. Participants responded via a button press that corresponded to “My sexual orientation *or* positive,” and another button press that corresponded to “Not my sexual orientation *or* negative.” Stage Four (10 trials) repeated Stage One, however the category labels were presented on the opposite side of the screen. Stage Five (20 trials) was similar to Stage Three, however one button press now corresponded to “My sexual orientation *or* negative,” and another button press corresponded to “Not my sexual orientation *or* positive.” Split-half reliability analyses indicated acceptable reliability (adjusted *r* = .60). Explicit–implicit affect ambivalence was calculated in the same manner as the SO ambivalence measure.^8^

#### Procedure

The study was conducted using DirectRT (Jarvis, 2008). Participants completed the explicit measure of SO prior to the ESE measure and then the explicit measure of affect toward one’s SO. Participants then completed the ISE measure before the implicit measures of SO and affect toward one’s orientation.

### Results

#### Descriptive statistics

##### Sexual orientation measures

As would be expected, straight participants were significantly more attracted to opposite-sex (*M* = 8.40, *SD* = .73) than same-sex individuals (*M* = 1.71, *SD* = .89), *t*(69) = 47.79, *p* < .0001, whereas gay participants were significantly more attracted to same-sex (*M* = 8.40, *SD* = .73) than opposite-sex individuals (*M* = 1.75, *SD* = .89), *t*(47) = −39.46, *p* < .0001.

In a 2 (straight/gay) × 2 (male/female) ANOVA, the implicit measure of SO revealed a significant main effect of SO, *F*(1, 114) = 315.27, *p* < .0001. Straight participants yielded an IAT effect indicative of a straight SO (*M*D’ = .67, *SD* = .42), whereas gay participants yielded an IAT effect indicative of a gay orientation (*M*D’ = −.77, *SD* = .33). The IAT effect for both straight and gay participants was statistically significant from zero (both *p*s < .0001), indicating that the critical trials were measuring a difference in valence.

The explicit and implicit measures were uncorrelated for both straight (*r* = .10) and gay participants (*r* = −.02).

##### Measures of self-esteem

The ESE measure did not reveal any differences between straight (*M* = 5.47, *SD* = 2.05) and gay participants (*M* = 5.73, *SD* = 1.95; *F* < 1). However, there were higher levels of indirectly measured self-esteem in gay (*M* = 7.83, *M* = 1.31) relative to straight participants (*M* = 6.80, *SD* = 1.53), *F*(1, 114) = 8.98, *p* = .003.

##### The congruency of ESE and ISE

Consistent with past research (Gebauer et al., 2008), there was a small but significant correlation between the self-esteem measures, *r* = .30, *p* = .001. As an exploratory exercise, the relationship between ESE and ISE was investigated by subtracting a participant’s standardized ISE score from their standardized ESE score. Under this conceptualization, a negative score refers to ISE being *higher* than ESE, whereas a positive score refers to ISE being *lower* than ESE. The former pattern reflects individuals with *damaged self-esteem*, whereas the latter pattern reflects individuals with *defensive self-esteem* (Jordan et al., 2009). A 2 (straight/gay) × 2 (male/female) ANOVA revealed that gay participants (*z* = −.22) had a significantly lower score on this index relative to straight participants (*z* = .15), *F*(1, 114) = 8.61, *p* = .004.

##### The importance of sexual orientation as a part of the self

A 2 (straight/gay) × 2 (male/female) ANOVA revealed a significant main effect of SO, *F*(1, 114) = 45.68, *p* < .0001. Overall, gay participants reported their SO as being more important to their sense of self (*M =* 6.13, *SD* = 1.48) compared with straight participants (*M* = 4.10, *SD* = 1.63).

##### Explicit measure of positive and negative affect toward one’s sexual orientation

This measure revealed no differences between straight (*M* = 7.73, *SD* = .98) and gay participants (*M* = 7.72, *SD* = 1.50; *F* < 1). However, both straight (*t*[69]) = 23.32, *p* < .0001) and gay participants (*t*[47] = 12.59, *p* < .0001) responded significantly higher than the scale mid-point, implying that participants reported positive affect about their SO.

##### Indirect measure of affect toward one’s sexual orientation

This measure revealed no differences between straight (*M*D’ = .53, *SD* = .42) and gay participants (*M*D’ = .51, *SD* = .48; *F* < 1). The D’ scores of both straight, *t*(69) = 10.71, *p* < .0001, and gay participants, *t*(47 = 7.41, *p* < .0001, were significantly greater than zero, indicating the largely positive attitudes held by all participants toward their orientations.

#### The impact of SO ambivalence on the time spent on explicit questions relating to sexuality: Straight participants

**Figure 4.**
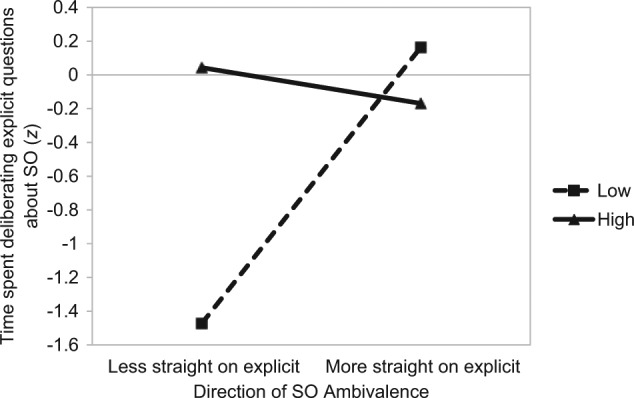
Study 3: The impact of the amount (separate lines) and direction (*x* axis) of SO ambivalence on time spent deliberating explicit questions on one’s sexuality (straight participants). *Note.* SO = sexual orientation.

A regression analysis revealed a marginally significant main effect of the amount of SO ambivalence on time spent deliberating on the explicit items, β = .29, *t*(66) = 1.78, *p* = .08. As in Studies 1 and 2, greater ambivalence was associated with longer deliberation. Replicating Studies 1 and 2, this main effect was qualified by the significant amount by direction interaction, β = −.62, *t*(66) = −2.78, *p =* .01 (see Figure 4). Like Studies 1 and 2, the interaction showed a clear distinction between self-identified straight individuals with low and high amounts of SO ambivalence *only* when individuals were *less straight* on the explicit measure of SO relative to the implicit measure. In this directional context, those with greater SO ambivalence spent significantly more time deliberating their SO relative to those with low amounts of SO ambivalence, β = .76, *t*(66) = 2.91, *p* = .01. Among individuals who were *more straight* on the explicit measure of SO relative to the implicit measure, there was no observable difference as a function of the amount of ambivalence, β = −.17, *t* < 1.

#### The impact of SO ambivalence on the time spent on explicit questions relating to sexuality: Gay participants

**Figure 5.**
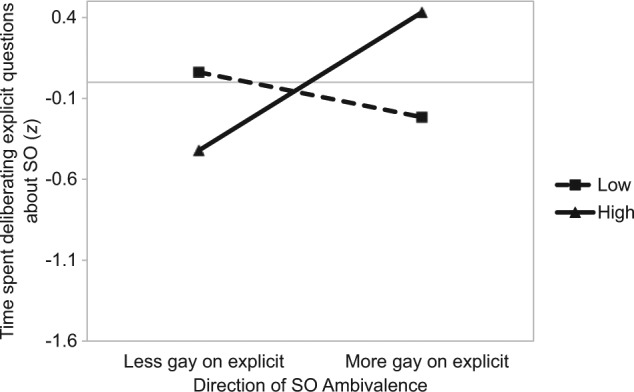
Study 3: The impact of the amount (separate lines) and direction (*x* axis) of SO ambivalence on time spent deliberating explicit questions on one’s sexuality (gay participants). *Note.* SO = sexual orientation.

Among gay participants, a regression analysis revealed a marginally significant amount by direction interaction, β = .41, *t*(44) = 1.81, *p* = .08. As shown in Figure 5, the pattern differs from that observed for straight participants. Here, we found a significant difference between those with high amounts of SO ambivalence as a function of direction. Individuals who were *more gay* on the explicit measure of SO relative to the implicit measure spent significantly more time deliberating their SO, β = .43, *t*(44) = 3.27, *p* = .002. No differences were observed between those with a low amount of SO ambivalence as a function of the direction of ambivalence, β = −.14, *t* < 1.

#### Implications of SO ambivalence for well-being: Straight participants

##### Self-esteem

Individual differences in the amount and the direction of SO ambivalence were not related to ESE, ISE, and the congruency between ESE and ISE (all *p*s > .10).

##### Centrality of SO and explicit affect toward SO

In regression analyses, we included individual differences in the amount and the direction of SO ambivalence and the respective interaction as the independent variables. We computed separate analyses for the outcomes of centrality and explicit affect. For centrality, the analysis revealed a significant main effect of the direction of SO ambivalence, β = −.50, *t*(66) = −2.17, *p* = .03. This was also found for explicit affect felt toward SO, β = −.43, *t*(66) = −1.86, *p* = .07. These findings suggest that individuals who reported being *more straight* on the explicit measure of SO relative to the implicit measure (i.e., those who experience implicit evaluations of their SO that are in conflict with self-reported identification) are more likely to be detached from and feel negative affect toward their SO. This is convergent with research showing the negative effects of concealment in addition to a difficulty of forming a positive identity of SO when concealment occurs (Frable et al., 1997; Hatzenbuehler et al., 2009).

#### Implications of SO ambivalence for well-being: Gay participants

##### Self-esteem

**Figure 6.**
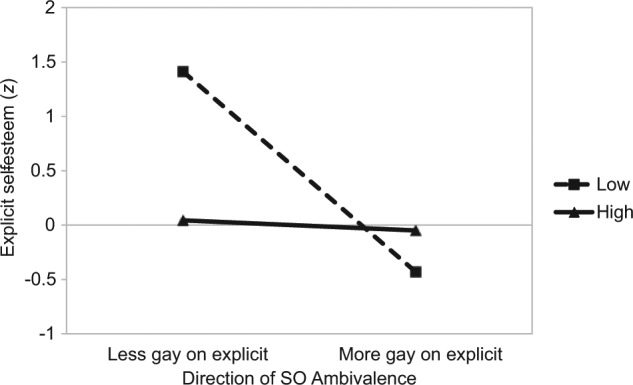
Study 3: The impact of the amount (separate lines) and direction (*x* axis) of SO ambivalence on explicit self-esteem (gay participants). *Note.* SO = sexual orientation.

On the explicit measure of self-esteem, among gay participants there was a significant amount by direction interaction, β = .62, *t*(44) = 2.61, *p* = .01 (see Figure 6). This showed that individuals with a low amount of SO ambivalence had the highest ESE when they reported being *less gay* on the explicit measure of SO relative to the implicit measure, β = −1.34, *t*(44) = −3.11, *p* = .003. Furthermore, a significant difference in ESE was observed for those with a low amount of SO ambivalence. Specifically, when participants reported being *less gay* on the explicit measure of SO relative to the implicit measure this implicated significantly higher self-esteem, β = −1.80, *t*(44) = 2.37, *p* = .02. For those individuals with a high amount of SO ambivalence, there was no observed impact of the direction of ambivalence on ESE, β = −.09, *t* < 1.

No significant effects were found on the ISE measure.

##### Centrality of SO and explicit affect felt toward SO

Individual differences in the amount and the direction of SO ambivalence were not related to the centrality of SO in addition of affect (all *p*s > .10).

#### Implications

of possessing discrepant E–I affective evaluations of sexual orientation on well-being

##### ESE–ISE congruency

When the ESE–ISE difference variable was entered into a linear regression model for gay participants, we found a significant main effect of the amount of ambivalence, β = .26, *t*(44) = 2.25, *p* = .03, such that a larger discrepancy between scores on the explicit and implicit measures of affect toward own SO resulted in ISE being lower than ESE. This suggests that higher affective ambivalence about an individual’s SO is associated with defensive self-esteem in gay participants.

This effect was not found among straight participants, β = −.04, *t* < 1.

### Discussion

Study 3 sought to replicate our previous findings and consider the effects of SO ambivalence among a sample of gay participants. In addition, we addressed the relationship between explicit–implicit SO ambivalence, well-being, and affective evaluations of SO.

Regarding the impact of SO ambivalence on deliberation, the primary findings among straight participants replicated the results of Studies 1 and 2. First, greater SO ambivalence was associated with longer deliberation to direct questions about sexuality. Second, when participants reported being *less straight* on the explicit measure of SO relative to the implicit measure, those with high ambivalence spent significantly more time deliberating relative to those with low ambivalence. Third, there was no observable difference in deliberation as a function of the amount of ambivalence when individuals reported being *more straight* on the explicit measure of SO relative to the implicit measure.

To our knowledge, this study provides the first evidence showing the importance of explicit–implicit SO ambivalence among gay individuals. Within our gay sample, a difference in deliberation as a function of the direction of SO ambivalence was observed *only* in those with high amounts of SO ambivalence. Specifically, significantly longer deliberation was seen among those who reported being *more gay* on the explicit measure of SO relative to the implicit measure (when compared with those who reported being *less gay* on the explicit measure of SO relative to the implicit measure). This might reflect a degree of identity conflict in these individuals; these individuals self-disclose as gay; however, their implicit evaluations of SO are somewhat incongruent with this perception. However, more research is necessary prior to making firm conclusions.

The study also investigated whether explicit–implicit SO ambivalence is related to well-being for both straight and gay participants. There is evidence that concealment of SO is associated with negative mental health outcomes (Hatzenbuehler, 2009; Meyer, 2003) as well as a difficulty in forming a positive identity of SO when concealment occurs (Frable et al., 1997). As such, we reasoned that self-identified straight individuals in our sample who experience conflicting explicit–implicit evaluations of their SO may experience negative outcomes. Consistent with this idea, among those who reported being *more straight* on the explicit measure of SO relative to the implicit measure, SO was found to be more detached from one’s sense of self. Moreover, these individuals reported more negative affect toward their SO.

When considering well-being among gay participants, the results revealed significantly higher self-esteem among those who reported being *less gay* on the explicit measure of SO relative to the implicit measure (when SO ambivalence was low). This effect might be explained by two reasons. First, given that prejudice toward gay individuals is still widespread (Herek & McLemore, 2013), direct responses that minimize one’s orientation could be adaptive in light of such prejudice. Second, low ambivalence represents less conflict between explicit and implicit evaluations. It is plausible that the combination of these two factors could result in higher levels of self-esteem.

Finally, we also found that a large explicit–implicit discrepancy in affective feelings toward SO was associated with defensive self-esteem in gay individuals. Future research could investigate whether such individuals are more defensive when it comes to their SO.

## General Discussion

Research on explicit–implicit attitudinal ambivalence has found that ambivalent individuals devote more attention to information that is relevant to their ambivalence (Briñol et al., 2006; Rydell et al., 2008). The present research investigated the consequences associated with explicit–implicit SO ambivalence. Study 1 investigated the relationship between individual differences in explicit–implicit SO ambivalence and the time spent deliberating direct questions about one’s SO. Study 2 replicated Study 1 and further demonstrated the robustness of Study 1’s effects by incorporating a different outcome measure. Study 3 extended the findings by using a sample of straight and gay men and women, and investigated as a secondary aim the implications of ambivalence for psychological well-being.

### Sexual Orientation Ambivalence and Processing

In all three studies, among self-identified straight participants, it was found that higher amounts of SO ambivalence resulted in more time spent deliberating direct questions about SO. This finding is consistent with extant research on explicit–implicit ambivalence (Briñol et al., 2006; Rydell et al., 2008). These effects also build upon research that addressed the implications of explicit ambivalence on information processing (e.g., Clark et al., 2008; Jonas et al., 1997; Maio et al., 1996).

One novel aspect of the present research is that this main effect was qualified across three samples (of straight participants) by an interaction between the amount and the direction of SO ambivalence. Among self-identified straight participants, this consistently revealed two key findings. First, for those who reported being *less straight* on the explicit measure of SO relative to the implicit measure, there was a significant difference in thinking about sexuality as a function of the amount of ambivalence. Second, when considering those who reported being *more straight* on the explicit measure of SO relative to the implicit measure, no differences in deliberation about SO were observed as a function of the amount of ambivalence. Taken together, these highly replicable findings unequivocally showed the important role of both the amount *and* the direction of ambivalence for the deliberation on SO among self-identified straight individuals.

The robustness of these findings was confirmed by using an alternative measure of deliberation. Specifically, among self-reported straight individuals who reported being less straight on the explicit measure of SO relative to the implicit measure, higher ambivalence was associated with greater elaboration of topic relevant information compared with those with low amounts of ambivalence. What might underlie this effect? On the basis of evidence that has shown ambivalence to result in greater elaboration of thoughts which might then help attenuate the negative effects of ambivalence (Nordgren et al., 2006), these findings imply that those with high amounts of SO ambivalence in this directional context elaborated more to resolve the underlying conflict. As such, we believe that in this directional context, the amount of ambivalence motivates deliberation and the subsequent resolution of ambivalence, in a way that converges with past research (Briñol et al., 2006; Rydell et al., 2008).

When considering those who reported being *more straight* on the explicit measure of SO relative to the implicit measure, individuals with low and high amounts of SO ambivalence demonstrated similar amounts of elaboration. What might underlie this effect? In this directional context, the basic implication could be explained by this group of self-identified straight individuals concealing some elements of same-sex identity that are assessed by the implicit measure of SO. These implicitly measured evaluations are potentially in conflict with self-identified SO. The very nature of this identity conflict (i.e., I report being straight, but I also have some identification with being gay) might be sufficient to produce effects that override the importance of the magnitude of explicit–implicit ambivalence. That is, the mere presence of such a conflict may be enough to want to reduce these effects via deliberation. This could potentially be the result of this identity (i.e., I have some identification with being gay) being one that still possesses a great deal of social stigmatization (Herek & McLemore, 2013).

Among gay participants, our findings on the deliberation to direct questions about sexuality revealed a different pattern of results. Specifically, a difference in deliberation as a function of the direction of SO ambivalence was observed *only* in those with high amounts of SO ambivalence. This revealed significantly longer deliberation among those who reported being *more gay* on the explicit measure of SO relative to the implicit measure. This might be explained by a high degree of identity conflict in such individuals; among self-reported gay individuals with high amounts of SO ambivalence, more ambivalence-relevant thinking occurs when individuals have an implicit evaluation of their SO suggesting some identification with being straight. Considering that such individuals have already “come out” as gay, it is understandable that such a situation would produce a strong motivation to focus on relevant information. This possibility is worthy of investigation in future research.

One question raised by the present findings regards why, in addition to a main effect of the amount of ambivalence, there is also an interactive effect of the amount and direction of ambivalence. As outlined above, we believe there is a good explanation underlying the pattern of effects found in this research. From our perspective, the current domain under investigation is more personally relevant compared with those used in previous research on explicit–implicit ambivalence. As such, it seems likely that different processes are involved when individuals consider topics that vary in personal relevance.

### Sexual Orientation Ambivalence and Well-Being

A secondary aim of the research was to begin to consider the link between SO ambivalence and well-being. Study 3 revealed that SO ambivalence was related to outcomes of psychological well-being in both straight and gay individuals. Among straight participants, greater detachment from SO and more negative affect was found among those who reported being more straight on the explicit measure relative to the implicit measure. Interestingly, this corresponds to the group of individuals who may experience identity conflict. Furthermore, these findings are consistent with research that has shown concealment of SO to result in negative psychological consequences (Hatzenbuehler, 2009; Meyer, 2003), in addition to concealment making it difficult to form a positive SO identity (Frable et al., 1997).

Furthermore, among gay participants, but not straight participants, ambivalence about SO was associated with feelings of self-worth in two distinct ways. First, significantly higher scores on the explicit measure of self-esteem were found among those who reported being less *gay* on the explicit measure of SO relative to the implicit measure, when the amount of SO ambivalence was low. This shows that SO ambivalence in some gay individuals, but not in others, is associated with improved psychological well-being. Second, when gay individuals had a large discrepancy between self-reported positive affect and indirectly measured positive affect toward SO, there were higher levels of defensive self-esteem. These findings complement previous work that has found gay individuals to be at greater risk for mental health problems (Haas et al., 2011; King et al., 2003, 2008; Meyer, 2003).

In sum, these findings begin to offer some interesting insights regarding the relation between explicit–implicit SO ambivalence and well-being. In straight participants, it is clear that there is an association between SO ambivalence and well-being when individuals are potentially concealing an identity conflict (i.e., same-sex attraction). In gay participants, the investigation of SO ambivalence provides a new and more focused direction for future research that can investigate other outcomes. In particular, given that SO ambivalence was associated with defensive self-esteem, it follows that SO ambivalence could also be associated with higher levels of out-group discrimination, nervousness, and impaired physical health (see, for example, Jordan, Spencer, & Zanna, 2005; Schröder-Abé, Rudolph, & Schütz, 2007).

## Conclusion

In all, this research makes a number of novel and important contributions in addition to providing interesting questions for future research. One point that is abundantly clear is that in the context of explicit–implicit SO ambivalence, both the amount and the direction of ambivalence are important when investigating how people process relevant information. In addition, the current research suggests that SO ambivalence produces different patterns of results in straight and gay individuals. Finally, the current research demonstrates that SO ambivalence is associated with indicators of well-being in both straight and gay participants.

## Notes

1. Please see Hofmann, Gawronski, Gschwendner, Le, and Schmitt (2005) for evidence regarding the lack of impact of block order on IAT scores and explicit–implicit measure order on their correlation.

2. The explicit–implicit relationship was assessed using a combined explicit sexual orientation (SO) score that encompassed attraction toward *both* men and women. We did this by subtracting the mean same-sex orientation (attraction and behavior toward women) from the mean opposite-sex orientation (attraction and behavior toward men). A higher score is therefore indicative of an opposite-sex orientation.

3. In all regression models reported in this article, the predictor variables were simultaneously entered.

4. The variance in discrepancy magnitude between those who were less gay versus more gay on the explicit measure of SO relative to the implicit measure was equivalent.

5. The efficacy of the manipulations was initially assessed by asking participants to rate the arguments in terms of strength on scale from 1 (very weak) to 9 (very strong). No difference in strength was found between the two editorials (*t* < 1). This is likely a product of the topic that is being studied. When collecting data for this study, the introduction of gay marriage in the United Kingdom was at the forefront of societal debate. Given the overwhelmingly positive attitudes of our participants toward the introduction of same-sex marriage, it is likely that any argument presented for the introduction of gay marriage would be seen as strong and compelling. Our findings show the expected differences between participants who read information that is *clearly relevant* to SO versus those who read information that *clearly is not* relevant to SO. As such, we are confident that this underlies the effect of our results.

6. While research has often used the total number of thoughts as an indication of the cognitive response, in the present study, such an outcome was too conservative. For instance, among participants who reported three thoughts, the number of words that were reported in the thoughts ranged from 12 to 120. As such, the number of words written was seen to more closely reflect the extent to which participants elaborated.

7. When the total number of thoughts was used as a dependent variable in the regression model, the analysis did not reveal an interaction between the amount and the direction of SO ambivalence. However, there was a significant interaction between the manipulation, the amount of ambivalence, and the direction of ambivalence, β = −.30, *t*(107) = −1.99, *p* = .05. This latter pattern is in line with that found when using the total number of words as the dependent variable.

8. Interestingly, the correlation between standardized scores on the explicit and implicit measures of affect toward one’s SO was non-significant, *r* = .01.
